# A Four-Methylated lncRNAs-Based Prognostic Signature for Hepatocellular Carcinoma

**DOI:** 10.3390/genes11080908

**Published:** 2020-08-08

**Authors:** Le-En Liao, Dan-Dan Hu, Yun Zheng

**Affiliations:** 1 State Key Laboratory of Oncology in South China, Collaborative Innovation Center for Cancer Medicine, Sun Yat-sen University Cancer Center, Guangzhou 510060, Guangdong, China; liaole@sysucc.org.cn (L.-E.L.); hudd@sysucc.org.cn (D.-D.H.); 2 Department of Colorectal Surgery, Sun Yat-sen University Cancer Center, Guangzhou 510060, Guangdong, China; 3 Department of Liver Surgery, Sun Yat-sen University Cancer Center, Guangzhou 510060, Guangdong, China

**Keywords:** hepatocellular carcinoma, lncRNAs, DNA methylation, survival analysis prognostic predictive model

## Abstract

Currently, an increasing number of studies suggest that long non-coding RNAs (lncRNAs) and methylation-regulated lncRNAs play a critical role in the pathogenesis of various cancers including hepatocellular carcinoma (HCC). Therefore, methylated differentially expressed lncRNAs (MDELs) may be critical biomarkers of HCC. In this study, 63 MDELs were identified by screening The Cancer Genome Atlas (TCGA) HCC lncRNAs expression data set and lncRNAs methylation data set. Based on univariate and multivariate survival analysis, four MDELs (AC025016.1, LINC01164, LINC01183 and LINC01269) were selected to construct the survival prognosis prediction model. Through the PI formula, the study indicates that our new prediction model performed well and is superior to the traditional staging method. At the same time, compared with the previous prediction models reported in the literature, the results of time-dependent receiver operating characteristic (ROC) curve analysis show that our 4-MDELs model predicted overall survival (OS) stability and provided better prognosis. In addition, we also applied the prognostic model to Cancer Cell Line Encyclopedia (CCLE) cell lines and classified different hepatoma cell lines through the model to evaluate the sensitivity of different hepatoma cell lines to different drugs. In conclusion, we have established a new risk scoring system to predict the prognosis, which may have a very important guiding significance for the individualized treatment of HCC patients.

## 1. Introduction

Hepatocellular carcinoma (HCC) accounts for 90% of primary liver cancer, which is one of the most common malignant tumors known and represents the second leading cause of cancer-caused deaths worldwide [1]. HCC is also the fourth most common cancer in China [2]. The main causes of HCC are chronic infection from the hepatitis virus, alcoholism, exposure to aflatoxin, smoking and diabetes [3]. The early diagnosis rate of HCC is relatively low, and most patients are diagnosed as advanced diseases. Although early diagnosis, imaging technology, surgical treatment, chemotherapy, molecular targeted therapy and other aspects of HCC have made great progress in recent years, the overall 5-year survival rate is still unsatisfactorily low [4]. Classic prognostic factors such as clinical lymph node metastasis (cTNM) stage and pathological stage (pTNM) are not fully prognostic in some patients [5,6]. Guidelines for prognostic assessment have been continuously modified to improve their accuracy and reduce the complexity of their daily clinical application [7,8]. Therefore, new effective and reliable prognosis and prediction biomarkers are needed to improve risk prediction and provide better information for guiding personalized treatment. Prognostic modelling is a useful tool in cancer management because it enables timely intervention in high-risk patients while avoiding unnecessary treatment in low-risk patients [9].

Non-coding RNAs (ncRNAs) function directly as structural, catalytic or regulatory RNAs, rather than encoding proteins [10,11]. According to their sizes, the regulatory ncRNAs can be classified as small ncRNAs (<200 bps, e.g., miRNAs, siRNAs, and piRNAs) and lncRNAs (>200 bps, e.g., lincRNAs, macroRNAs). LncRNAs have emerged as an essential regulator in almost all aspects of biology. Accumulating evidence suggests that lncRNAs play an important role in tumorigenesis [12]. Up to now, many studies have emphasized the molecular mechanism and biological characteristics of lncRNAs in the development of HCC [13,14]. Meanwhile, some lncRNAs may also be valuable prognostic predictors for HCC patients [15,16].

DNA methylation is a basic feature of epigenomes, which can affect the expression of protein-coding or non-coding transcripts [17,18]. In addition to the direct regulation of lncRNAs by DNA methylation through interactions with promoter regions, recent studies have also revealed some more complex regulatory relationships between lncRNAs and DNA methylation [19,20]. For example, the lncRNA ecCEBPA (extra-coding CEBPA) transcribed from the CEBPA gene locus has been reported as the key to regulate DNA methylation through interaction with DNA methyltransferase 1, DNMT1 at this site [21].The expression of lncRNAs maternally expressed gene 3 (MEG3) is related to the first intron methylation mediated by TET2 [22]. Furthermore, several studies have shown that lncRNAs is abnormal in HCC, which is associated with hepatocellular malignancy. Zheng et al., found a new lncRNA, named SRHC, which inhibits the proliferation of cancer and is partially down-regulated by DNA methylation in tumors [23]. Braconi’s research shows that HCC growth may be affected by methylation-dependent tissue-specific regulation of the lncRNAs MEG3 by miR-29a [24]. In addition, there is evidence that differentially expressed lncRNAs with altered methylation status may be an effective predictor of HCC [25,26]. However, a research linking DNA methylation, an essential disease biomarker and epigenetic regulator, with lncRNAs is still lacking. Therefore, in this study, we comprehensively analyzed the multidimensional data of TCGA and constructed a new predictive prognostic risk score system based on methylated differentially expressed lncRNAs (MDELs), which may be helpful for individualized treatment of HCC patients.

## 2. Materials and Methods

### 2.1. Data and Sources

The RNA sequencing data, DNA methylation data and corresponding clinical data of HCC and paired normal tissues were downloaded from TCGA (https://cancergenome.nih.gov/). There were 374 HCC samples and 50 paired normal tissues in total up to August 9, 2019. HCC cell line RNA-seq data and Medication data were downloaded from CCLE (https://portals.broadinstitute.org/ccle/ about).

### 2.2. Identification of Differential Expressed lncRNAs with Altered Methylation Status in HCC

**Figure 1:**
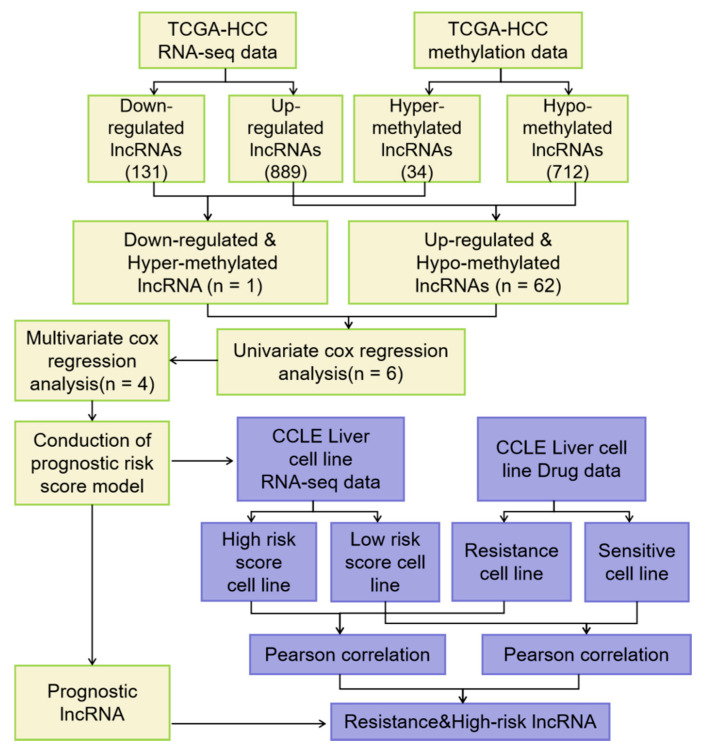
Flowchart of the study.

The expression data of lncRNAs were displayed as FPKM (fragments per kilobase of transcript per million mapped reads) and the expression level of each lncRNAs was normalizated by “EdgeR” package of R language for further analysis (EdgeR package, http://www.bioconductor.org/packages/release/bioc/html/edgeR.html). To obtain lncRNAs expression profile, we annotated lncRNA with the long non-coding RNA gene annotation file in GENCODE (Release 27). Next the differentially expressed lncRNAs were calculated by EdgeR (FDR (false discovery rate) < 0.05 and the absolute log2 FC > 1). The expression data set and DNA methylation data set were employed to identify the differentially expressed lncRNAs with altered methylation status. The flowchart of this study is shown in Figure 1. Firstly, EdgeR analysis was performed to identify the differential expressed lncRNAs (DELs) by comparing the normalized expression data between HCC and adjacent normal tissues. Next, we compared lncRNAs with altered methylation status from the TCGA data set and divided them into hyper-methylated lncRNAs and hypo-methylated lncRNAs (*t*-test, FDR < 0.05 and the absolute MTBeta-MNBeta > 0.3). These cutoffs and strategies were similar to those in previous studies [27,28]. Then we correlated the level of RNA expression with the degree of DNA methylation and in order to classify lncRNAs into two groups, hyper-methylated & down-regulated group and hypo-methylated & up-regulated group. LncRNAs of both groups were selected as candidate biomarker (MTBeta, MNBeta: β means of tumor samples and normal samples). Methylation levels were calculated as follows: We used the β value to estimate the methylation level of a given CpG probe. β value = Imeth/(Imeth + Iunmeth), where Imeth is the intensity of methylation and Iunmeth is the intensity of unmethylation [19,20].

### 2.3. Establishment of Prognostic Predictive Model and ROC Curve

The univariate Cox regression analysis was firstly performed on all of candidate lncRNAs to calculate the association between each lncRNAs expression and HCC patients’ OS. Only the lncRNAs with *p*-values less than 0.05 were identified as prognosis-related lncRNAs. Then, the selected key lncRNAs were further screened and confirmed by the multivariate Cox regression. The prognosis risk score model was established with the following formula:

Prognosis Index (PI) = expression of lncRNA 1 × β1 + expression of lncRNA 2 × β2 +… expression of lncRNA n × βn.

Briefly, we used multivariate cox regression analysis to get the prognosis index formula. The expression values of lncRNAs in different patients were input, and the risk coefficient was calculated by using the PI formula. Based on the cut-off the median PI, HCC patients were then divided into high- and low- risk score subgroups.

### 2.4. LncRNAs Survival Analysis

Univariate and multivariate Cox proportional hazards regression analyses were further performed to investigate the prognostic effect of this prognosis risk score, and comparison were made for Gender, Age, Tumor stage, Pathologic stage, Body mass index (BMI), Adjacent hepatic tissue inflammation, Fibrosis, Radiation therapy, Relative family cancer history, Pharmaceutical therapy, New tumor event after initial treatment, Person neoplasm cancer status and PI. The time-dependent ROC curve analysis within 5 years as the defining point was conducted with the R package “pROC” (pROC package version 1.15.3, https://cran.r-project.org/web/packages/pROC/index.html), to assess the predictive accuracy of prognostic model for time dependent disease outcomes. Kaplan-Meier survival curves were drawn to evaluate the relationship between all parameters (clinical inspects and prognosis risk score) and OS of HCC patients. ROC curve was applied to evaluate the predictive value of the risk score for patients’ outcome after first course of treatment.

### 2.5. Use Prognostic Risk Score Model to Evaluate HCC in Cell Line

According to the inhibitory concentration 50 (IC50) value of a certain drug, the cell lines were divided into drug-resistant and drug-sensitive cell lines. If the IC50 value of one drug in the cell line was greater than the mean value of all 20 drugs plus 0.8 times of the standard deviation, the cell line was considered to be resistant to the drug. Inversely, the cell line was considered to be sensitive to the drug. In terms of the risk coefficient, the cell lines were divided into high-risk and low-risk cell lines by using the expression data of HCC cell lines in CCLE and prognostic risk score model.

### 2.6. Statistical Analyses

The correlation between lncRNA expression and DNA methylation level was tested using Pearson correlation. The univariate and multivariate Cox regression analysis were performed with “survival” package (survival package version 2.44-1, https://cran.r-project.org/web/packages/survival/index.html). All statistical analyses were performed with R language version 3.6.0. All P values are two-sided with values less than 0.05 being considered statistically significant. C-index was calculated using the survival package’s function coxph. We compared different models by using the area under the curve (AUC) and concordance index (C-index), a value reflects the prognostic discrimination ability.

## 3. Results

### 3.1. Identification of DELs with Altered lncRNAs Methylation Status in HCC

**Figure 2:**
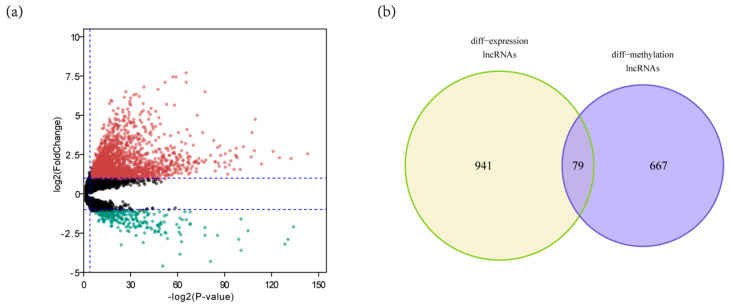
Identification of DELs and DMLs in HCC. (**a**) The DELs in HCC and normal tissues (*n* = 1020); (**b**) The intersection of 1020 DELs and 746 DMLs (*n* = 79).

**Figure 3:**
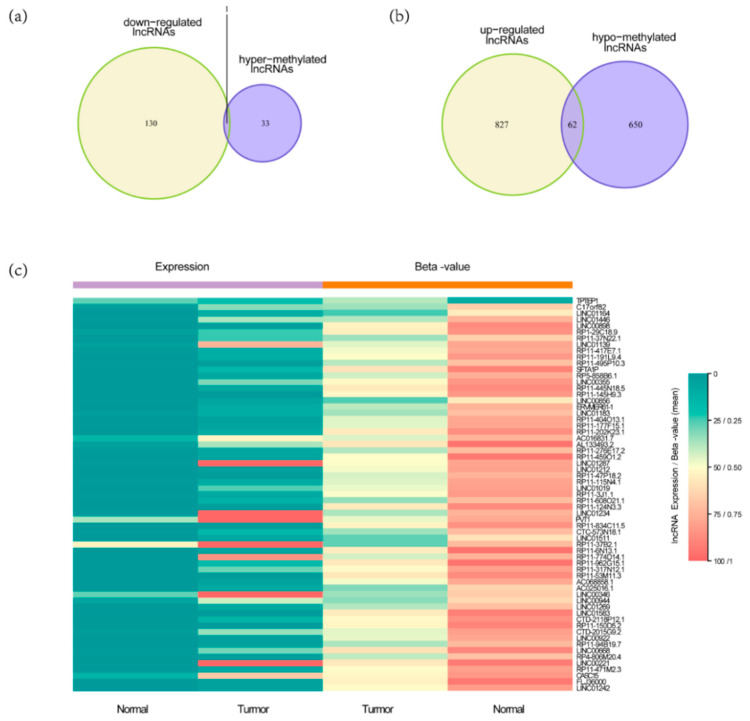
The process of screening candidate lncRNAs and their expression profiling in HCC tissues and normal tissues. (**a**) Downregulated lncRNAs with hypermethylation status (*n* = 1); (**b**) Upregulated lncRNAs with hypomethylation status (*n* = 62); (**c**) 63 lncRNAs expression and methylation cluster analysis.

Firstly, we used expression data from TCGA HCC RNA-seq data set for limma analysis to select DELs. A total of 1020 DELs were identified, including 131 down-regulated lncRNAs and 889 up-regulated lncRNAs (FDR < 0.05 and the absolute log2 FC (fold change) > 1) (Figure 2a). Then, we used methylation data from TCGA HCC methylation data set for T test to select differentially expressed lncRNAs with altered methylation status (DMLs). A total of 746 DMLs were identified, including 34 hypermethylated lncRNAs and 712 hypomethylated lncRNAs. The intersection of 1020 DELs and 746 DMLs was 79 (Figure 2b). By comparing with the lncRNAs methylation patterns of TCGA HCC methylation data set, we further identified one down regulated lncRNAs (Figure 3a) with hypermethylation and 62 up regulated lncRNAs with hypomethylation (Figure 3b). LncRNAs expression and methylation cluster analysis were carried out for the above 63 molecules (Figure 3c).

### 3.2. Construction of Four-MDELs HCC Prognostic Risk Score Model

**Figure 4:**
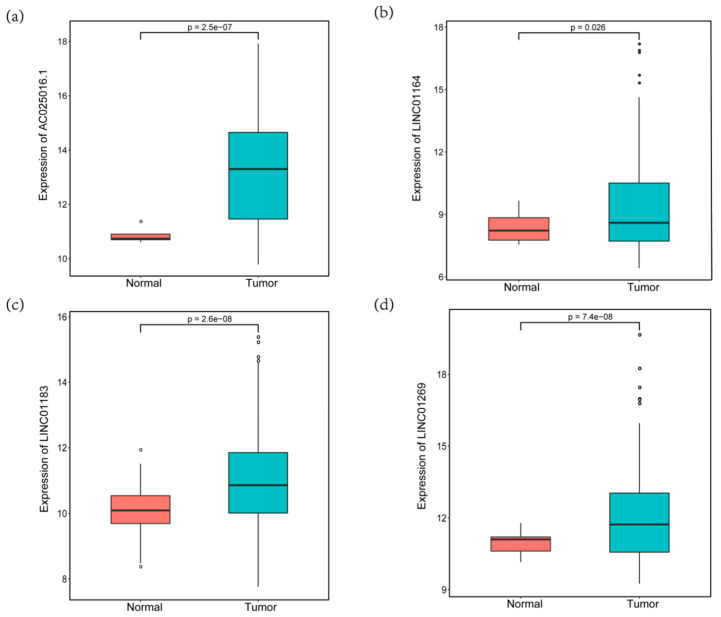
The expression of four hypomethylated lncRNAs in HCC and normal tissues. (**a**) AC025016.1, (**b**) LINC01164, (**c**) LINC01183; (**d**) LINC01269.

**Figure 5:**
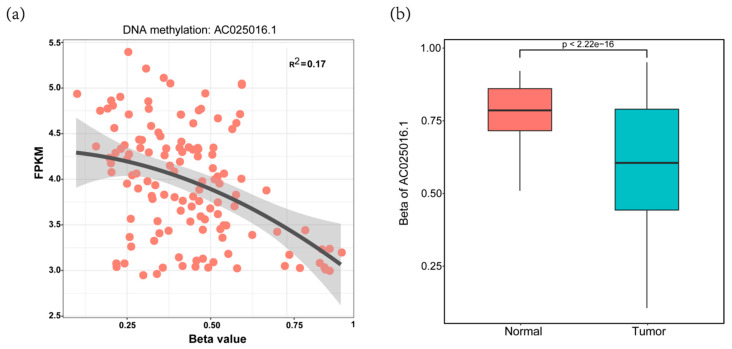
Example of a lncRNA driven by DNA methylation. (**a**) the AC025016.1 expression level (FPKM) was inversely correlated with its promoter methylation level (β value); (**b**) Boxplot showing that the β values of primary tumor samples were significantly higher than those of normal tissue samples.

Six prognosis-related lncRNAs (*p* < 0.05) were obtained through the univariate Cox regression analysis of the 63 candidate lncRNAs and the survival data of the discovery cohort. Through the multivariate Cox regression analysis of the 6 lncRNAs, we obtained an optimal combination (AC025016.1, LINC01164, LINC01183 and LINC01269, Table 1). These four lncRNAs were significantly over expressed in HCC tissues, indicating that they may be potential biomarkers in HCC patients (Figure 4a–d). Some lncRNAs expression levels in tumor samples were probably regulated by DNA methylation. As an example, we showed that the expression level of AC025016.1 was slightly inversely correlated with the DNA methylation level at its promoter region (R2 = 0.170) (Figure 5a). This suggests methylation leads to decreased lncRNA expression. The promoter region of AC025016.1 was hypomethylated in tumor samples (Figure 5b). Based on multivariate Cox regression analysis, the risk score formula we got from the discovery cohort was:

PI = 0.0031 × AC025016.1 expression + 0.0021 × LINC01164 expression + 0.0130 × LINC01183 expression+0.0064 × LINC01269 expression.

### 3.3. Validation and Assessment of Four-MDELs HCC Signature Prognostic Risk Score Model

**Figure 6:**
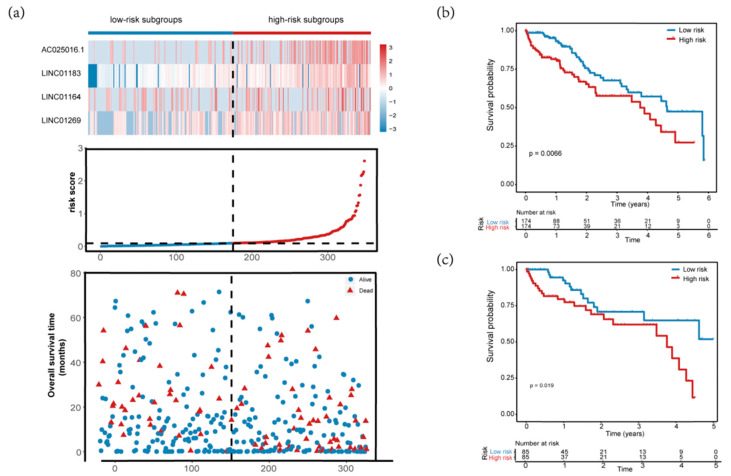

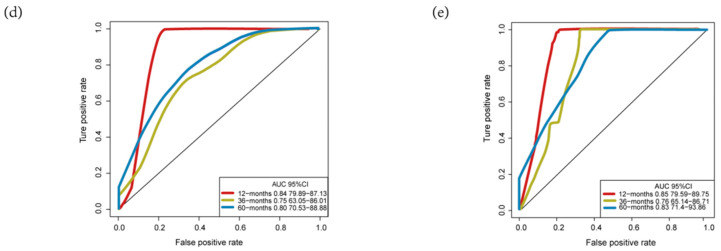
Assessment of four-MDELs HCC signature prognostic risk score model. The patients were stratified into high risk group and low risk group based on median of risk score. (**a**) Risk score distribution of HCC patients, Survival status of each patient and Expression heatmap of the four-MDELs corresponding to each sample above. Red: high expression and risk; Blue: low expression and risk; (**b**,**c**) Kaplan-Meier estimates of the OS time of the patients in the training set and the validation set using the four-MDELs signature-based risk score; (**d**,**e**) The ROC curve of the training set and validation set for 1-,3- and 5-year survival prediction.

In order to validate the applicability and prognostic value of the prognostic risk score model we constructed based on the entire TCGA data set, we randomly divided the 348 HCC patients in the entire data set into a validation set (*n* = 174). According to the formula, we calculated each patient’s risk score, and the patients in the training set and the validation set were divided into high- and low-risk groups based on the median value of the risk score. The distribution of risk score, survival status and expression spectrum of MDELs in four kinds of prognosis are shown in Figure 6a. In addition, we conducted analysis using a Kaplan-Meier curve to evaluate the effect of risk score on patients’ OS time. 

The Kaplan-Meier curves indicated that the median survival time of patients of high-risk group was 3.76 years, far shorter than that of low-risk group 4.64 years. Our results showed that patients with high risk score had significantly poorer OS than patients with low risk score (*p* = 0.0066, Figure 6b). Consistent with the results observed in the entire data set, the prognosis of the high-risk group was worse than that of the low-risk group in the validation set (*p* = 0.019, Figure 6c). The ROC curves of the training set and validation set also show good performance. The AUCs for the 1-year, 3-years, and 5-years OS of the training set were 0.84, 0.75 and 0.8, respectively (Figure 6d). The AUCs for the 1-year, 3-years and 5-years OS of the validation set were 0.85, 0.76, and 0.83, respectively (Figure 6e).

### 3.4. Comparisons with Other Models

**Figure 7:**
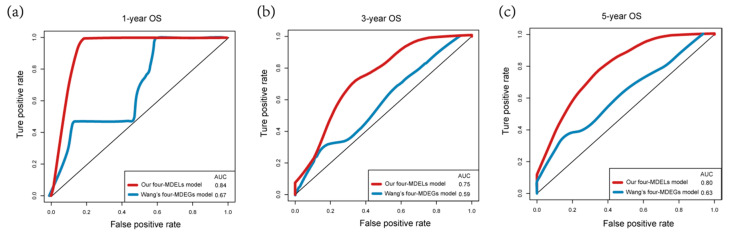
Comparison of our four-MDELs model and other literature model. The red and blue lines represent the ROC curve of the four-MDEL mode and Wang’s four-MDEG model respectively for (**a**) 1-year (**b**) 3-year (**c**) 5-year survival prediction.

Recently, Wang et al., reported a model containing four methylated differently expressed genes based on four genomics profiling data sets (GSE62232, GSE84402, GSE73003 and GSE57956) [29]. In order to compare the prognostic values of the four-MDELs model with their model, we conducted a time-dependent ROC curve analysis. The results showed that Wang’s model predictive value obviously decreased in predicting 1-, 3- and 5- years OS whereas the four-MDELs model was stable and much better in predicting prognosis (Figure 7, Table 2). We also compared their C-index values. The C-index statistical value of four-MDELs signature is 0.61 (95% CI: 0.55–0.67), which is greater than Wang’s model 0.58 (95% CI: 0.52–0.64). Considering the clinical characteristics of patients, including gender, age, T stage, pathologic stage, adjacent hepatic tissue inflammation, virus, fibrosis, radiation therapy, relative family cancer history, and person neoplasm cancer status, univariate and multivariate Cox regression analysis were used to evaluate the characteristics of OS (high-risk vs. low-risk). In the univariate analysis, virus (HBV or HCV infection) (HR = 0.649, *p* = 0.042), T stage (HR = 1.794, *p* = 0.011), relative family cancer history (HR= 1.795, *p* = 0.009), person neoplasm cancer status (HR = 2.208, *p* = 0.009) and the four-MDELs signature (HR = 1.838, *p* = 0.007) were all significantly associated with OS in HCC patients. However, only PI remained statistically significant with the confirmation by multivariate analysis (HR = 2.469, *p* = 0.010, Table 3). Because of its good stability and robustness, the four-MDELs model was selected as the ideal one.

### 3.5. Application of Our Model in HCC Cell Lines

**Figure 8:**
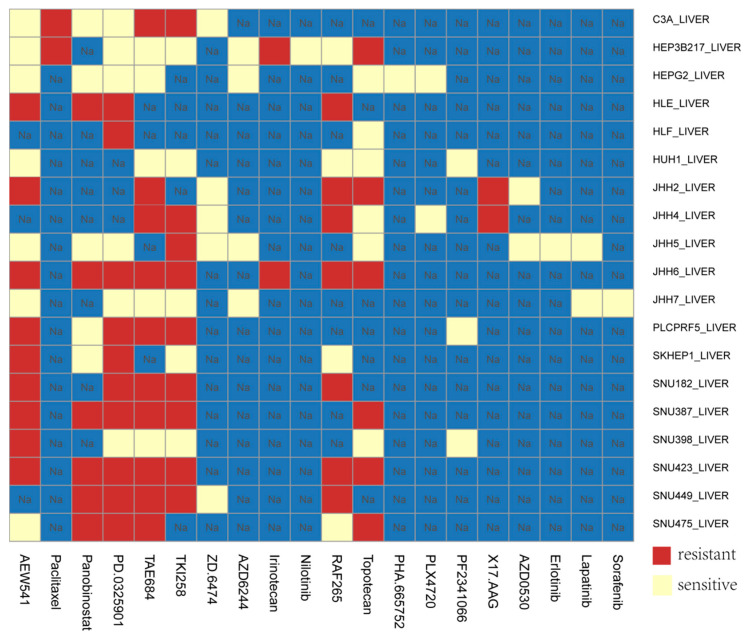
Application of prognostic risk score model in HCC cells.

Due to the lack of clinical medication data of HCC patients, we applied the prognostic risk score model in CCLE HCC cell lines. The model was used to evaluate the sensitivity of different HCC cell lines to different drugs. According to the risk coefficient, combined with the expression data of hepatoma cell lines in CCLE, we can divide the cell lines into high-risk and low-risk cell lines. The results showed that in JHH7 and SNU182 cell lines, the risk score was positively correlated with the IC50 of different drugs (Pearson correlation coefficient: 0.9, *p*-value: 4.536 × 10^−5^), that is, the high-risk cell lines tended to be resistant, while the low-risk cell lines were more sensitive to drugs (Figure 8).

## 4. Discussion and Conclusions

HCC is the fifth most common form of cancer worldwide and the third most common cause of cancer-related deaths [30]. Due to the lack of effective and reliable prognostic biomarkers or models, the clinical prognosis of HCC patients remains a major target for improvement. Over the past decade, a large number of studies have shown that lncRNAs plays a critical role in the process of tumor development [31,32]. Furthermore, methylation has also come to be recognized as an important epigenetic regulator of gene expression in eukaryotes, and it is now well established that methylation is crucially involved in multiple cancers, including HCC [33,34]. Few studies have combined the two fields. To our knowledge, this is the first study to develop a MDELs-based risk score that is predictive of prognosis in HCC.

Over the past few decades, many studies have explored prognostic models for HCC patients. In the present study, we mined the public data set of lncRNAs methylation and expression profile from TCGA and searched for a new prognostic marker for HCC. Interestingly, we found 79 MDELs in HCC (Supplementary Table S1). We observed the limited overlap between the DMLs and DELs. Differential expression of lncRNAs does not always correlate with methylation levels. This could be due to the facts that the differentially expressed lncRNAs levels in the cells are controlled by multiple factors, including binding of transcription factors to the corresponding promoters; epigenetic regulation such as DNA methylation and histone modification; and RNA stability such as modification of 5′ or 3′ UTRs. Some of these lncRNAs have been recognized as being associated with cancer. For example, C17orf82 was reported to be significantly overexpressed in HBV-related HCC [35]. LncRNA LINC01446 promotes glioblastoma progression by modulating miR-489-3p/TPT1 axis [36]. Based on the above 79 MDELs, we found that the PI constructed by 4-MDELs had good prognostic value for HCC.

Through univariate and multivariate survival analysis, we determined that 4-MDELs had the ability to independently predict the OS of patients. We then developed a risk score model based on four prognostic lncRNAs expressions. The risk model was used to divide the patients into low risk group (LRG) and high-risk group (HRG). LRG patients are more likely to survive longer than HRG patients. In addition, in the univariate analysis, virus (HBV or HCV infection) (HR = 0.649, *p* = 0.042), T stage (HR = 1.794, *p* = 0.011), relative family cancer history (HR = 1.795, *p* = 0.009), person neoplasm cancer status (HR = 2.208, *p* = 0.009) and the four-MDELs signature (HR = 1.838, *p* = 0.007) were all significantly associated with OS in HCC patients. However, only PI remained statistically significant with the confirmation by multivariate analysis (HR = 2.469, *p* = 0.01, Table 2). The results showed that our risk score model was more relevant to liver cancer patients’ survival than clinical factors such as viral infection, pathological staging and person neoplasm cancer status. Meanwhile, in order to evaluate the prognostic value of the four-MDELs model, we compared the model with other studies. There were many models for studying differential methylation of DNA or differential expression of lncRNAs, but there is still a lack of research linking DNA methylation with lncRNAs expression. For instance, Zhao et al., constructed a robust microRNA (miRNA)-based signature for predicting head and neck squamous cell carcinoma (HNSCC) prognosis use expression data [37]. Shen’s team established 11 differential lncRNAs expression prognostic model for breast cancer [38]. The methylation of the BRCA1 promoter was associated with a poor patient outcome [39]. The analysis of DNA differential methylation models can assess the damage status of tumor patients’ genomes from the perspective of epigenetics, while the lncRNAs differential expression model can evaluate the damage status of tumor patients’ genomes from the transcriptome level. In our study, we comprehensively considered the two factors and evaluated the damage status of tumor patients’ genomes from multiple dimensions. We believed that our results were more comprehensive and accurate. Such as, We chose Wang’s model containing four methylated differently expressed genes [29]. The time-dependent ROC curve shows that the Wang’s model predictive value obviously decreased compared with our model in predicting 1-, 3- and 5- years OS and the four-MDELs model is stable and much better in predicting prognosis (Figure 7).

To confirm our results, we first explored the expression profile of the above-mentioned lncRNAs in other data sets and databases but did not find a suitable cohort to verify our survival prediction model. Considering that many microarrays were not designed for lncRNAs, we did not find a suitable data set in GEO or other database, which included methylation data and expression profile of these four lncRNAs as well as detailed clinical survival analysis data. Therefore, we only compared the expression of these four prognostic lncRNAs in normal tissues and tumors using data from the TCGA database. At the same time, there are very few reports on the above four lncRNAs. Shi’s et al., study showed that lncRNA AC025016.1 was significantly differentially expressed in lung cancer, with a fold change value of 55.84 [40]. Huang’s et al., study found that lncRNA LINC01269 may have a very important biological function, which is related to the OS rate and disease-free survival rate of patients with esophageal squamous cell carcinoma [41]. Han’s et al., study found that LINC01183 was the 10 most downregulated lncRNAs in gastric cancer cells [42]. In addition, The LncRNA and Disease Database (version 2.0) predicts that LINC01164 and LINC01269 are associated with a variety of cancers, such as non-small cell lung cancer and gastric cancer. Especially LINC01269, which is probably related to liver cancer [43]. Studies have shown that these lncRNAs are differentially expressed in a variety of tumor patients and normal people, which may be related to the prognosis of tumor patients and can be used as a molecular marker to evaluate the prognosis of tumor. We were also interested in the future molecular mechanisms of these four lncRNAs. Unfortunately, these lncRNAs have not been published, and little is known about the mechanism of action of these four lncRNAs.

Finally, we used this prognostic risk score model in CCLE HCC cells lines. The results showed that in JHH7 and SNU182 cell lines, the risk score was positively correlated with the IC50 of different drugs, that is, the high-risk cell lines tended to be resistant, while the low-risk cell lines were more sensitive to drugs. For instance, TKI258 (dovitinib) is a multi-target inhibitor, which can inhibit VEGFR, PDGFR, FGFR and other targets. The target drug has a wide range of applications in liver cancer. For drug TKI258 in CCLE data, the IC50 values of cell lines JHH7 and SNU182 were 1.727μM and 8μM. Through the analysis of the model, the JHH7 have a low risk (0.106) for the target drug TKI258 and the SNU182 have a high risk (0.328), that is, the JHH7 cell line is sensitive to TKI258, while the SNU182 is resistant to TKI258. This result was therefore consistent with the data in CCLE (Figure 8). In the future, combined with the clinical data of patients, this model can be applied to clinical practice. It can be used not only to evaluate and predict the survival prognosis of patients with different liver cancers, but also to classify patients and evaluate and predict the sensitivity of different groups of patients to different treatment drugs.

Inevitably, our study has some limitations. First, due to the limited clinical data of patients, we could not conduct another subgroup analysis and verification through more factors. Second, the prediction model was built and evaluated based on common data sets. We hoped to obtain more comprehensive data in the future and develop more accurate prognostic evaluation models. In addition, more experiments were needed to further verify the clinical and biological functions of these four lncRNAs.

In conclusion, we identified 63 differentially expressed lncRNAs with altered methylation and selected four lncRNAs (AC025016.1, LINC01183, LINC01164 and LINC01269) to build a prognosis model. Survival analysis showed that our risk score model had significant prognostic value for HCC patients. Compared with American Joint Committee on Cancer (AJCC) staging and other literature models, our model has a great advantage in predicting the stability and accuracy of prognosis, which is expected to be applied to the clinical prognosis assessment of HCC patients.

## Figures and Tables

**Table 1:** 4-lncRNAs’ differential methylation region or site.

Name	Chr	Start	End
AC025016.1	chr11	5,944,985	5,949,571
LINC01183	chr5	127,802,442	127,802,442
LINC01269	chr14	70,707,575	70,707,575
LINC01164	chr10	131,773,701	131,773,701

Note: The reference genome version used for the above analysis was hg38.

**Table 2:** Comparison of our model and other literature models.

OS	AUC (Our Model)	AUC (Wang’s Model)
1-year	0.84	0.67
3-year	0.75	0.59
5-year	0.80	0.63

**Table 3:** Univariate and multivariate Cox regression analysis of characteristics and prognosis index in HCC.

Characteristics	Patients	Univariate Analysis		Multivariate Analysis	
		HR ^1^ (95% CI) ^2^	*p*–Value	HR (95% CI)	*p*–Value
**Gender (male/female)**	255/122	0.758 (0.498–1.154)	0.196	0.763 (0.482–1.208)	0.249
Age (≤ 65 vs. > 65)	235/141	0.666 (0.441––1.007)	0.054	0.729 (0.463–1.145)	0.17
Tumor (T3–T4/T1–T2)	94/280	1.794 (1.146–2.808)	0.011	0.715 (0.086–5.919)	0.756
Pathologic stage (III–IV/I–II)	91/262	1.483 (0.906–2.427)	0.117	0.744 (0.298–1.856)	0.526
BMI ^3^ (≥ 25 vs. <25)	161/180	0.906 (0.577–1.423)	0.669		
Adjacent_hepatic_tissue_inflammation (no/yes)	119/120	1.128 (0.662–1.923)	0.657		
Virus ^4^ (no/yes)	211/166	0.649 (0.428–0.985)	0.042		
Fibrosis (no/yes)	76/142	0.813 (0.474–1.397)	0.454		
Radiation therapy (no/yes)	243/4	2.127 (0.29–15.622)	0.458		
Relative_family_cancer_history (no/yes)	212/114	1.795 (1.16–2.779)	0.009		
Pharmaceutical_therapy (no/yes)	103/26	2.189 (0.951–5.04)	0.066		
New tumor event after initial treatment (no/yes)	149/142	1.592 (0.877–2.89)	0.126	0.654 (0.179–2.382)	0.519
Person neoplasm cancer status (with tumor/tumor free)	123/157	2.208 (1.224–3.983)	0.009	2.816 (0.774–10.245)	0.116
PI (High–risk/Low–risk)	174/174	1.838 (1.177–2.87)	0.007	2.469 (1.238–4.923)	0.01

^1^ hazard ratio; ^2^ 95% confidence interval; ^3^ body mass index; ^4^ HBV or HCV infection.

## Supplementary Materials

The following are available online at https://www.mdpi.com/2073-4425/11/8/908/s1, Table S1: Methylated differential expressed lncRNAs.
